# Global Analysis of Lysine Lactylation of Germinated Seeds in Wheat

**DOI:** 10.3390/ijms242216195

**Published:** 2023-11-11

**Authors:** Junke Zhu, Weiwei Guo, Yubin Lan

**Affiliations:** 1 School of Agricultural Engineering and Food Science, Shandong University of Technology, Zibo 255000, China; zhujunke@sdut.edu.cn; 2 College of Agronomy, Qingdao Agricultural University/Shandong Key Laboratory of Dryland Farming Technology/Shandong Engineering Research Center of Germplasm, Innovation and Utilization of Salt-Tolerant Crops, Qingdao 266109, China; 3 National Sub-Center for International Collaboration Research on Precision Agricultural Aviation Pesticide Spraying Technology, Shandong University of Technology, Zibo 255000, China; 4 Academy of Ecological Unmanned Farm, Shandong University of Technology, Zibo 255000, China

**Keywords:** posttranslational modification, lactylome, seed germination, wheat

## Abstract

Protein lactylation is a newly discovered posttranslational modification (PTM) and is involved in multiple biological processes, both in mammalian cells and rice grains. However, the function of lysine lactylation remains unexplored in wheat. In this study, we performed the first comparative proteomes and lysine lactylomes during seed germination of wheat. In total, 8000 proteins and 927 lactylated sites in 394 proteins were identified at 0 and 12 h after imbibition (HAI). Functional enrichment analysis showed that glycolysis- and TCA-cycle-related proteins were significantly enriched, and more differentially lactylated proteins were enriched in up-regulated lactylated proteins at 12 HAI vs. 0 HAI through the KEGG pathway and protein domain enrichment analysis compared to down-regulated lactylated proteins. Meanwhile, ten particularly preferred amino acids near lactylation sites were found in the embryos of germinated seeds: AA*K^la^T, A***K^la^D********A, K^la^A**T****K, K******A*K^la^, K*K^la^********K, K^la^A******A, K^la^*A, KD****K^la^, K********K^la^ and K^la^G. These results supplied a comprehensive profile of lysine lactylation of wheat and indicated that protein lysine lactylation played important functions in several biological processes.

## 1. Introduction

Epigenetic regulation often occurs to proteins during or after protein biosynthesis and plays vital roles in multiple biological processes. Post-translational modifications (PTMs) could change the function of proteins through adding new groups, for example, acetyl, methyl, phospho, ubiquityl, succinyl and lactyl groups. Among these PTMs, lysine lactylation was a reversible and dynamic process by the enzymatic reaction catalyzed by p300 enzyme [1,2].

As one of the novel post-translational modifications, lysine lactylation (Kla) was recently found to occur both on histone and non-nuclear proteins [3]. Lactate was often regarded as the product of glycolysis and provided energy for many development processes [4]. The concentrations of intracellular lactate could affect the Kla’s level, which is related to glycolysis [3]. Histone lactylation, much like histone acetylation, influences the expression of genes by changing the relationship between histones and DNA [1]. 

Wheat (*Triticum aestivum* L.), an important cereal crop in the world, could provide energy for 30% of the world’s population [5]. Previous proteomics analysis showed that many PTMs participate in diverse biological processes in wheat, such as lysine acetylation and lysine succinylation [6,7]. Seed germination, as the first stage of wheat development, could affect grain yield directly [8]. At the same time, the process is often strictly regulated by environmental and endogenous signaling pathways. Guo et al. found that lysine acetylation might play a crucial role in wheat seed germination, and there are 1,301 acetylated sites on 722 proteins during the germination stage [9]. Meanwhile, lysine acetylation, malonylation, succinylation and 2-hydroxyisobutyrylation widely participate in developing rice seeds [10,11,12,13]. In the latest study, lysine lactylation was reported to be involved in the development of rice grains [3]. However, the mechanisms of protein lactylation that regulate seed germination are still unclear. Herein, our study performed the first global lactylome profile in wheat germinated seeds, which is meaningful to agriculture science. 

## 2. Results

### 2.1. Global Profiling of Proteome in Embryos of Wheat at Different Hours after Imbibition 

**Figure 1:**
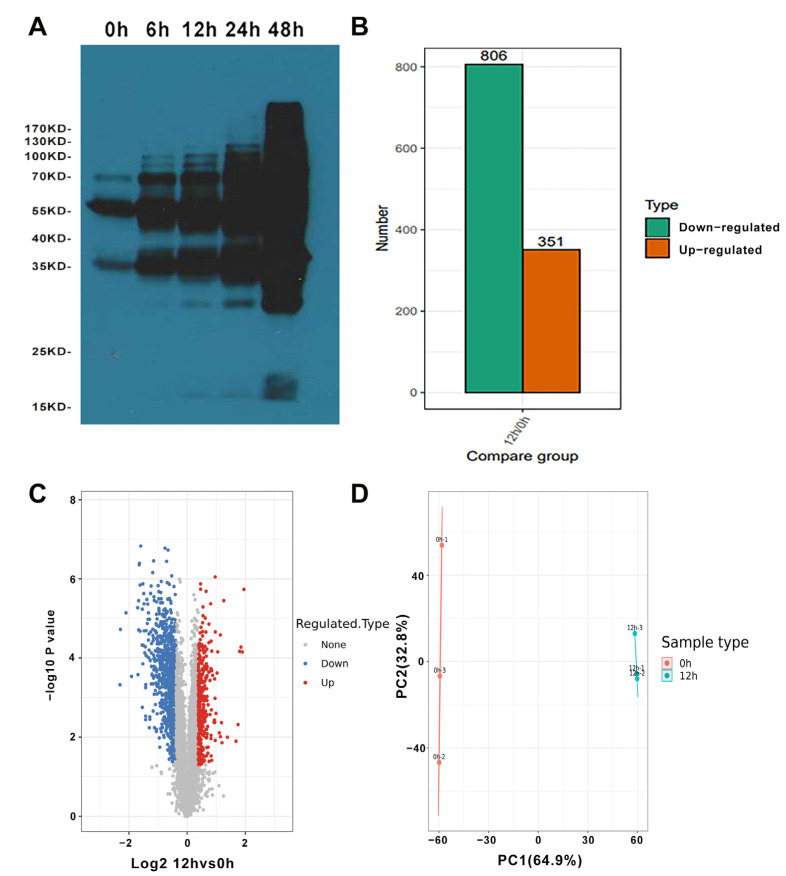
Identification of lysine lactylation in wheat germinated seeds. (**A**) Lysine lactylation profile in germinated wheat seed of QiMin 23 revealed by western blotting. (**B**) The differentially expressed proteins (DEPs) of germinated wheat seed in 12 HAI vs. 0 HAI. (**C**) The volcano plot analysis of 0 h vs. 12 h. The *y*-axis denotes the P-value. The *x*-axis denotes the fold change. (**D**) The PCA analysis of germinated seed samples at 0 h and 12 h.

In order to uncover the regulated mechanisms of germinated seeds in wheat at the proteome and lactylome level, the embryos of 0, 6, 12, 24 and 48 HAI were used for lactylation signals analysis by SDS-PAGE (Figure S1) and western blotting (Figure 1A), and the bands were divergent as the time progressed, suggesting that lysine lactylation is a specific distribution at different periods of germinated wheat seeds. Based on the result, 0 HAI and 12 HAI were selected for further analysis.

In total, 8000 proteins were identified, and 7433 proteins were quantified (Table S1). The differentially expressed proteins (DEPs) were screened between 0 and 12 HAI (Table S2), with the threshold changed >1.5 and *p* < 0.05. Compared with 0 HAI, there were 1157 DEPs (351 up-regulated and 806 down-regulated) in 12 HAI (Figure 1B,C). In order to test the conditions and the replicates’ integrity, we performed PCA analysis using the total protein abundance of all samples, and the result showed that the repeatability of different protein samples was consistent (Figure 1D). 

### 2.2. Functional Enrichment Analysis of the DEPs

**Figure 2:**
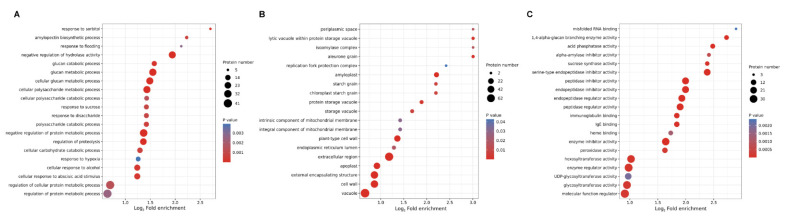
GO-based enrichment analysis of differentially expressed proteins (DEPs) in 12 HAI vs. 0 HAI. The *y*-axis denotes the categories of (**A**) biological process, (**B**) cell component and (**C**) molecular function. The *x*-axis denotes the fold enrichment.

In order to investigate the characteristics of the DEPs, GO enrichment-based clustering analysis was displayed among the category of cellular components, biological processes and molecular functions (Figure 2). The results showed that numerous proteins that were related to processes such as the response to sorbitol, amylopectin biosynthetic process, response to flooding and negative regulation of hydrolase activity were enriched at 12 HAI in the biological process (Figure 2A). Meanwhile, proteins related to processes involving more periplasmic space, lytic vacuole within protein storage vacuole, isoamylase complex and aleurone grain were enriched at 12 HAI treatment in the cellular component analysis (Figure 2B). Consistently, compared with 0 HAI, proteins related to processes involving misfolded RNA binding, 1,4-alpha-glucan branching enzyme activity, acid phosphatase activity, alpha-amylase inhibitor activity, sucrose synthase activity and serine-type endopeptidase inhibitor activity were significantly enriched at 12 HAI in the molecular function analysis (Figure 2C). 

**Figure 3:**
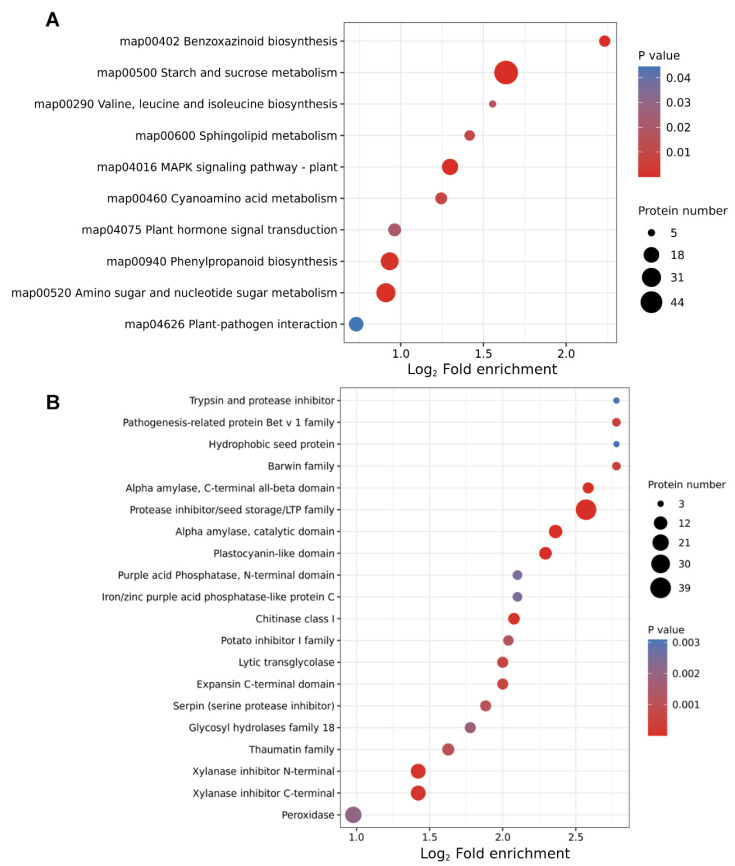
KEGG pathway and protein domain enrichment analysis of differentially expressed proteins (DEPs) in 12 HAI vs. 0 HAI. The *y*-axis denotes the categories of (**A**) KEGG pathways and (**B**) protein domains. The *x*-axis denotes the fold enrichment.

The KEGG pathway enrichment analysis revealed a significant enrichment of proteins related to the benzoxazinoid biosynthesis process at 12 HAI (Figure 3A). Meanwhile, compared with 0 HAI, many diverse metabolism pathways, such as pathways related to starch and sucrose, valine, leucine and isoleucine biosynthesis and sphingolipid metabolism, were dramatically enriched at 12 HAI, which was consistent with previous studies [9]. In order to investigate the distribution of the DEP in different germination stages, protein domain analysis was performed (Figure 3B). The results showed that trypsin and protease inhibitor, pathogenesis-related protein Bet v 1 family, hydrophobic seed protein and barwin family proteins were more notably enriched at 12 HAI compared to 0 HAI (Figure 3B).

### 2.3. Proteome-Wide Analysis of Lysine Lactylation Sites and Proteins in Germinated Seeds of Wheat

**Figure 4:**
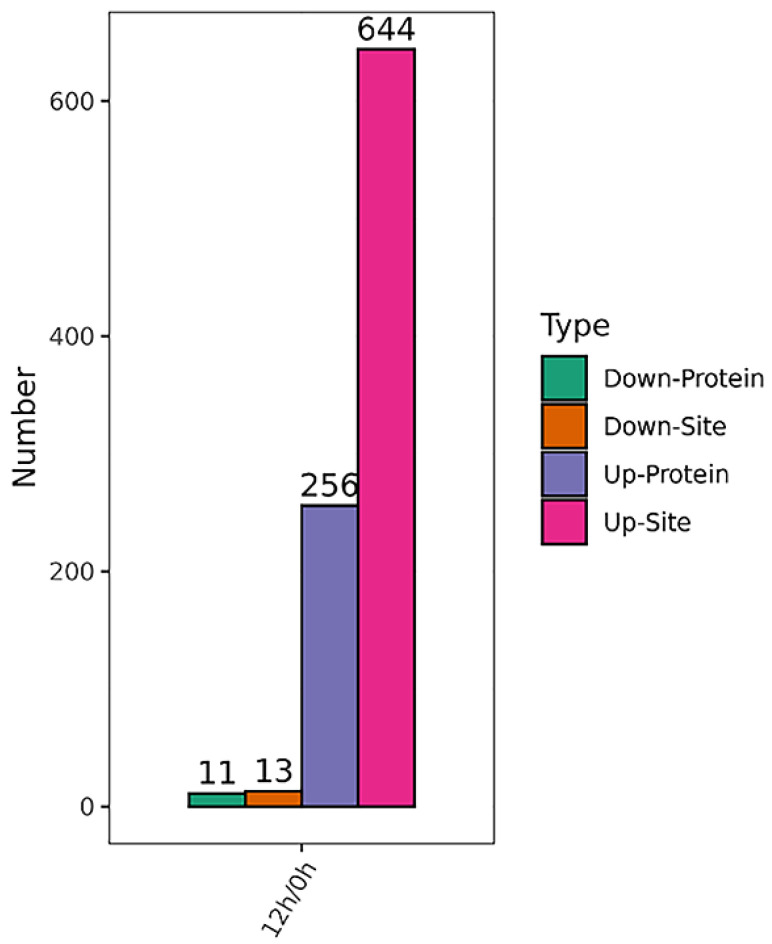
The differentially lactylated proteins and sites of germinated wheat seeds in 12 HAI vs. 0 HAI. The *y*-axis denotes the number of each type. The *x*-axis denotes the up or down protein and site.

Lysine lactylation, one modification of lysine residue, is expected to play a key role in wheat metabolism and development. However, it has barely been studied in wheat seeds until now. In our study, 927 lactylated sites in 394 proteins were identified, among which 821 sites in 337 proteins were accurately quantified (Table S3). The differentially lactylated proteins (DLPs) and differentially lactylated sites (DLSs) in wheat embryos between 0 and 12 HAI were analyzed under the threshold change fold > 1.3 and *p* < 0.05 (Table S4). More up-regulated lactylated proteins and sites were identified in 12 HAI vs. 0 HAI group (644 sites in 256 proteins), while less lactylated proteins and sites showed a decreased lactylation level at 12 HAI (13 sites in 11 proteins) (Figure 4).

### 2.4. Characterization of DLPs in Wheat Germinated Seeds

**Figure 5:**
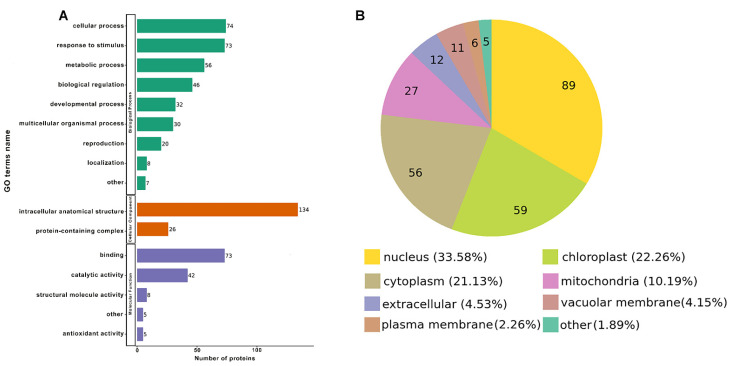
Functional classification of the differentially lactylated proteins (DLPs) in germinated wheat seed. (**A**) Classification of the lactylated proteins based on Gene Ontology. (**B**) Subcellular localization of the lactylated proteins. Note: The data of the pie chart denotes the number of each item.

To better identify the potential roles of lysine lactylation in germinated seeds of wheat, all the differentially lactylated proteins (DLPs) were used in Gene ontology (GO) classification analysis based on the biological process, cellular component and molecular function. The results showed that most of the DLPs were classed into cellular process (21%) and response to stimulus (21%) in the “biological process” category of the GO classification (Figure 5A). In the classification of the GO term “cellular component”, 84% of the DLPs were in an intracellular anatomical structure (Figure 5A). Meanwhile, in the classification of the GO term “molecular function”, most up-regulated Kla-modified proteins were classified into the binding category (55%) (Figure 5A).

The subcellular localization of lactylated proteins was also analyzed. The results showed that most of the proteins were distributed in the nucleus (33.58%), chloroplast (22.26%), and cytoplasm (21.13%), respectively (Figure 5B). Together with the GO functional classification data, all the results indicated that lactylated proteins might participate in diverse biological processes in germinated seeds of wheat.

### 2.5. Functional Enrichment Analysis of DLPs

**Figure 6:**
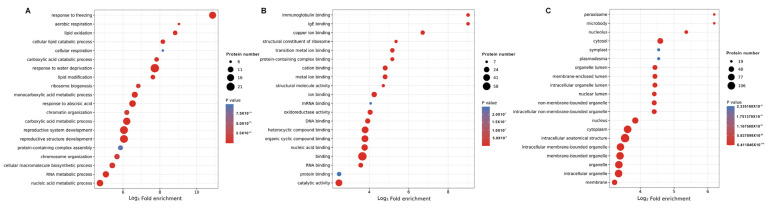
GO-based enrichment analysis of the differentially lactylated proteins (DLPs) in 12 HAI vs. 0 HAI. The *y*-axis denotes the categories of (**A**) biological process, (**B**) molecular function and (**C**) cell component. The *x*-axis denotes the fold enrichment.

In order to investigate the categories of DLPs in wheat germinated seeds, GO enrichment (biological process, molecular function and cellular component), KEGG pathway enrichment and protein domain analyses were performed. In relation to the response to freezing process, various metabolic processes and development-related processes, the DLPs were greatly enriched according to biological process enrichment (Figure 6A). The results of molecular function enrichment analysis showed that many lactylated proteins were associated with binding activities in the 12 HAI vs. 0 HAI group (Figure 6B). In agreement with these findings, DLPs were more enriched in peroxisome, microbody, nucleolus and cytosol in 12 HAI vs. 0 HAI based on the cellular component enrichment analysis (Figure 6C). 

**Figure 7:**
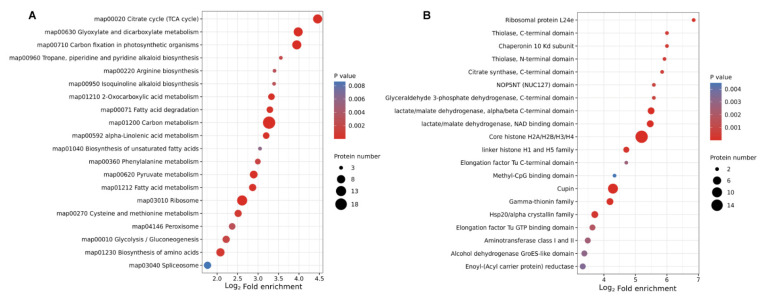
KEGG pathway and protein domain enrichment analysis of the differentially lactylated proteins (DLPs) in 12 HAI vs. 0 HAI. The *y*-axis denotes the categories of (**A**) KEGG pathways and (**B**) protein domains. The *x*-axis denotes the fold enrichment.

Consistent with the observations, the KEGG pathway enrichment analysis also demonstrated that proteins related to the TCA cycle, metabolism and biosynthesis tended to be lactylated in the 12 HAI vs. 0 HAI group (Figure 7A). Meanwhile, the results of the protein domain showed that the central-metabolism-related proteins were significantly lactylated (Figure 7B). 

### 2.6. Motif Analysis of Lysine Lactylated Peptides

**Figure 8:**
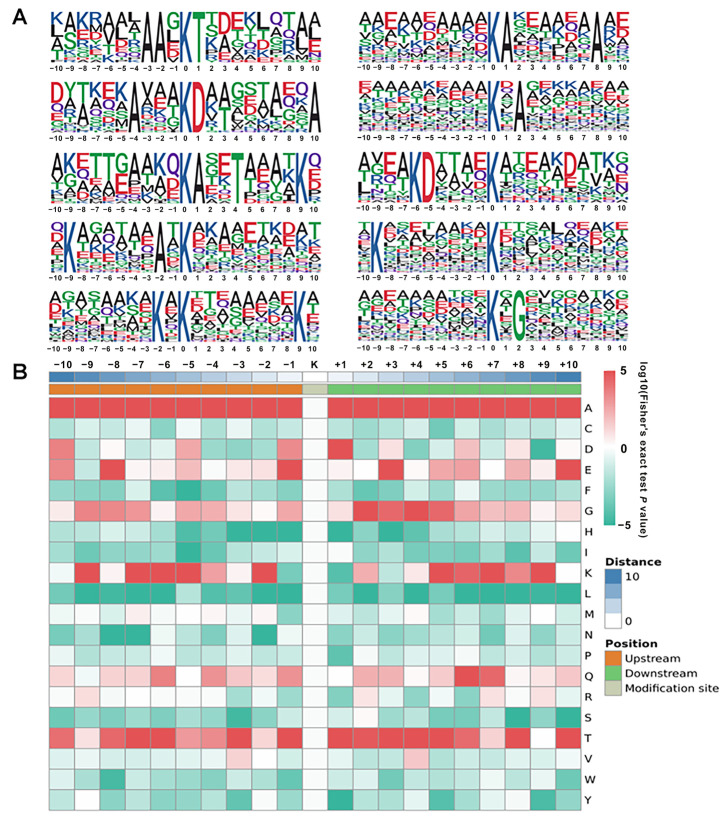
Properties of lysine lactylated peptides. (**A**) Lactylation sequence motifs for ±10 amino acids surrounding the lysine lactylation sites. (**B**) Probability sequence motifs of lactylation sites consisting of 10 residues surrounding the targeted lysine residue using Motif−X. The different colors in the figure show the frequency changes of each amino acid at a specified position. Red denotes high frequency, and green denotes low frequency.

In order to evaluate the properties of lactylated sites in germinated seeds of wheat, the surrounding amino acid sequence was analyzed by Motif-X program. A total of 10 consensus sequence motifs were enriched, including AA*K^la^T, A***K^la^D********A, K^la^A**T****K, K******A*K^la^, K*K^la^********K, K^la^A******A, K^la^*A, KD****K^la^, K********K^la^ and K^la^G (K^la^ represents the lactylated lysine, and * indicates a random amino acid residue) (Figure 8A). Consistent with the observations, the results of the heat map showed that the frequency of alanine (A) was highest in the +1 and −1 positions near lactylation sites, indicating that it was a preferred amino acid surrounding the lactylation sites (Figure 8B). Meanwhile, aspartic acid (D) and threonine (T) were also significantly overrepresented in the −1 and +1 positions, which was consistent with the four identified motifs (Figure 8B). In summary, motif analysis supplied a better way to understand the potential role of Kla-modified peptides.

## 3. Discussion

Lysine lactylation, as a novel PTM, has recently been identified in mice, humans and rice [1,3]. The process could be stimulated by lactate, which was derived exogenously or endogenously [14]. In our study, we displayed a widespread lysine lactylation analysis of germinated seeds in wheat. In the germinated seeds of wheat, a total of 927 lactylated sites in 394 proteins were identified, and 821 sites in 337 proteins were quantified (Table S3). Lysine lactylation was also identified in humans, mice, *B. cinerea* and rice [1,3,15]. The results of the characterization of differentially lactylated proteins showed that DLPs were distributed to multiple functional groups and localized in diverse cellular compartments (Figure 5), which was consistent with rice [3]. All these results indicate that lysine lactylation plays an important role in the development of wheat seed.

As a cereal crop in the world, wheat plays an important role in ensuring food security [16]. Thus, the quality of seed germination could directly affect the level of wheat yield [3]. During the seed germination process, much energy is required [17]. In this research, we found that the DLPs were more enriched in various metabolism-related processes according to GO enrichment (Figure 6A), and many lactylated proteins were enriched in the central metabolism, which could transform carbon into energy by glycolysis and the TCA cycle (Figure 7). Further analysis showed that most DLPs were enriched in up-regulated lactylated proteins at 12 HAI vs. 0 HAI based on the KEGG pathway and protein domain enrichment analysis (Tables S5 and S6). A similar result was also found in rice grains [3]. All these studies suggested that lysine lactylation might participate in the regulation of the metabolism process in the germinated seeds of wheat.

As the first stage of wheat growth and development, seeds could be subjected to biological and abiotic stresses during germination. Meanwhile, seeds could activate a series of mechanisms in response to these challenges [18]. In our study, we found 10 lactylated sites in 6 stress-related proteins at 12 HAI vs. 0 HAI (Table S4), suggesting that lysine lactylation could be involved in the regulation of stress-related proteins during seed germination. Moreover, the 70-kD heat shock protein (HSP70) was also found to be lactylated, which was previously identified as being associated with stress [19,20]. This result suggested that the HSP70 protein may play vital roles in wheat seed germination.

## 4. Materials and Methods

### 4.1. Plant Material and Growth Conditions 

QiMin 23, the common wheat variety (*Triticum aestivum* L.), was used in the study. The seeds were washed three times in distilled water and then imbibed in a dark growth chamber at 21 °C. The embryos were respectively collected 0, 6, 12, 24 and 48 h after imbibition (HAI) with three biological replicates and were then snap-frozen for 1 min with liquid nitrogen and stored at −80 °C for further western blotting, proteome or acetylome analysis.

### 4.2. Protein Extraction and Western Blotting

Proteins of QiMin 23 embryos were extracted with a modified phenol isolation protocol [21,22]. In brief, embryos were ground into fine powder in liquid nitrogen and homogenized in lysis buffer (0.5 M Tris 8.0, 1 M sucrose, 50 mM ascorbic acid, 0.1 M KCl, 1% NaDOC, 1% NP40, 10 mM EDTA, 10 mM DTT, and 1% protease inhibitor cocktail; the final pH was 8.0). The resulting solution was sonicated on ice for 30 min and then an equal volume of tris-saturated phenol (pH 8.0) was added. The remaining debris were depleted by centrifugation for 10 min at 16,000× *g*. Next, the supernatant was added to four volumes of precipitation buffer (methanol with 0.1 M ammonium acetate, precooled at −20 °C before use) and stored at −20 °C overnight. The pellet was collected though centrifugation at 16,000× *g* for 10 min and was then rinsed with cold methanol three times. A total of 0.4 mL lysis buffer was added to the final protein pellet, and the protein concentration was estimated with the 2D Quant kit (GE Healthcare, Chicago, IL, USA).

For the western blotting analysis, 30 μg protein samples was electrophoresed in 12% SDS-PAGE gel, and Kla-modified proteins were detected in a standard procedure.

### 4.3. Trypsin Digestion

A total of 4 mg proteins was first reduced using 5mM DTT for 30 min at 56 °C before being alkylated with 30 mM iodoacetamide (IAM) for 15 min. After suspension in 0.1 M, the resulting proteins were digested with sequencing grade trypsin (Promega V5280, Madison, WI, USA) at a trypsin: substrate ratio of 1:50 (w/w) overnight. The peptides were purified by Strata X C18 SPE column (Phenomenex, Torrance, CA, USA) and vacuum-dried [23].

### 4.4. TMT Labelling

A total of 2 mg peptides was labelled with a TMT kit under the manufacturer’s instruction [6]. In brief, peptides were resuspended using 50 mM HEPES. Each TMT reagent, which was first dissolved in acetonitrile, was mixed with a peptide sample and then incubated for 2 h at room temperature. Subsequently, the mixture was pooled, desalted and vacuum-dried. 

### 4.5. Affinity Enrichment of Lys-lactylated Peptides

The peptides were dissolved in NETN buffer (100 mM NaCl, 1 mM EDTA, 50 mM Tris-HCl, 0.5% NP-40, pH 8.0) and then incubated with prewashed anti-lactyllysine antibody-conjugated agarose beads (Micrometer Biotech, Hangzhou, China) overnight with gentle shaking at 4 °C. Subsequently, the beads were washed four times with NETN buffer and twice with deionized water. Kla-modified peptides were eluted with 0.1% trifluoroacetic acid (TFA) and cleaned with C18 ZipTips (Millipore, MA, USA).

### 4.6. HPLC-MS/MS Analysis

After desalting with C18 ZipTips, the enriched lactylated peptides (about 0.1–1 ug) were dissolved in 0.1% formic acid and then separated using a C18 capillary column (15 cm length, 75 μm i.d.) on an EASY-nLC 1000 UPLC system [24]. The peptides were ionized and electrosprayed into the mass spectrometer and detected using the Orbitrap at a resolution of 70,000 (m/z 200) with an NCE setting of 30. The range of m/z was set from 350 to 1800 for the MA scan [7,25]. LC-MS/MS analysis was performed blindly by the Micrometer Biotech Company (Hangzhou, China).

### 4.7. Database Search

The obtained MS/MS raw data of lactylated peptides were processed by MaxQuant (v.1.5.2.8) with the integrated Andromeda search engine (v.1.5.2.8) [7]. Meanwhile, TMT-6plex was used for quantitative analysis. The tandem mass spectra were searched against the UniProt_Triticum database (146,090 sequences released March 2015) concatenated with a reverse decoy database [26]. The parameters in MaxQuant were performed according to Zhang et al. [7]. 

Mass error was set to 10 ppm for precursor ions and 0.02 Da for fragment ions. Carbamidomethyl on Cys was specified as a fixed modification, and oxidation on Met, deamidation (NQ), Klactyl (H(4) O(2) C(3):72.021Da) and Acetyl(Protein N-term) were specified as variable modifications. The minimum peptide length was set to 7. All the other parameters in MaxQuant were set as default values. The site localization probability was set as >0.75.

### 4.8. Bioinformatics Analyses

The Gene Ontology (GO) annotation was derived from the UniProt-GOA database (http://www.ebi.ac.uk/GOA/) [7]. The Kyoto Encyclopedia of Genes and Genomes (KEGG) database and InterProScan were used to annotate the protein pathway and protein domains, respectively. Meanwhile, the WoLF PSORT databases were used to annotate subcellular localization. Soft motif-X was used to analyze the motif of lysine lactylation sites. Enrichment-based clustering analysis was set with R-package (v.2.0.3) following the procedure described previously [19].

The two-tailed Fisher’s exact test was used to examine the enrichment of identified proteins against all proteins in the database during GO, KEGG, and domain enrichment analysis. Correction for multiple hypothesis testing was performed using standard false discovery rate control methods. All the terms with adjusted *p*-values below 0.05 in any of the clusters were considered significant.

## 5. Conclusions

In summary, our study provided the first extensive data on lysine lactylation in wheat germinated seeds. A total of 8000 proteins and 927 lactylated sites were identified at 0 and 12 HAI. Meanwhile, some particularly preferred amino acids were found near lactylation sites, including AA*K^la^T, A***K^la^D********A, K^la^A**T****K, K******A*K^la^, K*K^la^********K, K^la^A******A, K^la^*A, KD****K^la^, K********K^la^ and K^la^G, in the germinated seeds of wheat. Further analysis showed that more up-regulated and down-regulated lactylated proteins were enriched at 12 HAI vs. 0 HAI based on the KEGG pathway and protein domain enrichment analysis, respectively. Some glycolysis- and TCA-cycle-related proteins were found with lactylation changes. All the results confirmed the notion that lysine lactylation played critical regulatory roles in multiple aspects of germinated seeds. Thus, this study might provide important resources for further functional analysis of lysine lactylation in germinated seeds of wheat. 

## Data Availability

All the data presented in this study are included in the manuscript and Supplemental Materials. The mass spectrometry raw data have been deposited to ProteomeXchange Consortium via the PRIDE with the dataset identifier PXD046488.

## Supplementary Materials

The supporting information can be downloaded at: https://www.mdpi.com/article/10.3390/ijms242216195/s1.
