# Supplementary material for: Global Analysis of Lysine Lactylation of Germinated Seeds in Wheat
Source: Int J Mol Sci. 2023 Nov 11;24(22):16195. doi: 10.3390/ijms242216195 (PMC10671351; doi:10.3390/ijms242216195)
Supplement: Supplementary file 1 [file ijms-24-16195-s001.zip › Figure S1.pdf]

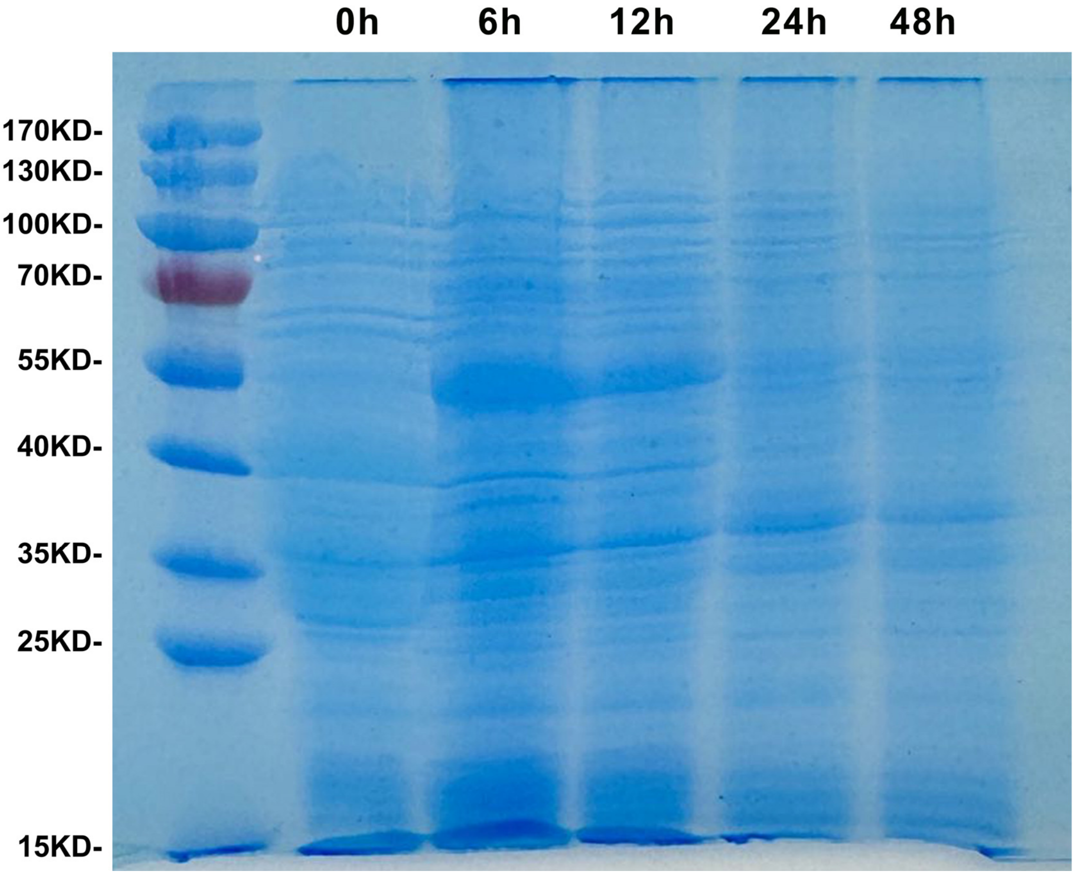

Supplementary Figure S1. The SDS-PAGE gel electrophoresis of embryo at different time points after wheat seed germination.
